# Meta-analysis of the therapeutic effects of various methods for the treatment of chronic atrial fibrillation

**DOI:** 10.3892/etm.2013.1158

**Published:** 2013-06-14

**Authors:** GANG LIN, HUI-HE LU, YI SHEN, JIAN-FEI HUANG, LIN-SHENG SHI, YI-NAN GUO

**Affiliations:** 1 Cardiovascular Medical Department, The First People’s Hospital of Nantong, Nantong, Jiangsu 226001;; 2 School of Public Health, Nantong University, Nantong, Jiangsu 226009, P.R. China

**Keywords:** chronic atrial fibrillation, radiofrequency catheter ablation, drug therapy

## Abstract

The aim of this study was to analyze the therapeutic effects of various methods for the treatment of chronic atrial fibrillation (AF). Randomized controlled trials (RCT) concerning drug therapy and catheter ablation for the treatment of chronic AF were retrieved. The RevMan 5.1 software package was used for the meta-analysis. A total of 20 papers were assessed in this study. The results of the analysis indicated that the success rate was lower [odds ratio (OR), 8.94; 95% confidence interval (CI), 4.70–17.02; P<0.0001] and the relapse rate was higher (OR, 0.07, 95% CI, 0.05–0.10; P<0.0001) for drug therapy compared with that for catheter ablation. With regard to different catheter ablation procedures, the success rate for pulmonary vein antrum isolation (PVAI) was lower compared with that for PVAI plus complex fractionated atrial electrogram (CFAE; OR, 0.53; 95% CI, 0.37–0.78; P=0.0001). Pulmonary vein isolation (PVI) plus left atrial ablation (LAA) had a higher success rate compared with PVI alone (OR, 2.79; 95% CI, 1.59–4.88, P=0.0003). There was not identified to be a significant difference in the success rates between PVAI and CFAE (OR, 2.05; 95% CI, 0.06–205.74; P=0.76) or between PVI and circumferential pulmonary vein isolation (CPVI; OR, 0.94; 95% CI, 0.29–3.00; P=0.91). All the funnel plots of publication bias were essentially symmetrical. In conclusion, the success rate was higher and the relapse rate was lower for catheter ablation compared with drug therapy. Among the different procedures of catheter ablation, there were no significant differences in success rate between two single procedures; however, the success rates were higher for the combined methods compared with those for the single methods.

## Introduction

Atrial fibrillation (AF) is the most common tachyarrhythmia (1). The incidence of AF is 3–5% in individuals aged >65 years and 9% in individuals aged >80 years (2). The incidence of cerebral apoplexy and heart failure is increased in patients with AF since the heart atrium loses mechanical function with slow blood flow (3,4). AF is the strongest independent risk factor for cerebral apoplexy and heart failure; 15% of cerebral apoplexy and 30% of heart failure cases are associated with AF and the mortality rate of patients with AF is three times the mortality rate of patients with sinus rhythm (5). Compared with other types of AF, chronic persistent AF is more complex and is difficult to treat. According to the AF treatment guidelines produced by the American College of Cardiology, American Heart Association and European Society of Cardiology (ACC/AHA/ESC) in 2006, chronic AF is defined as AF that persistently exists following drug therapy or electroversion (6).

A great deal of attention has been paid to radiofrequency catheter ablation for the treatment of chronic AF. The relapse rate of radiofrequency catheter ablation is 20–60% in the treatment of chronic AF (7–9). At present, there is no standard radiofrequency catheter ablation method for the treatment of chronic AF. The main methods of radiofrequency catheter ablation for treating chronic AF include pulmonary vein isolation (PVI), pulmonary vein antrum isolation (PVAI), circumferential pulmonary vein isolation (CPVI), complex fractionated atrial electrogram (CFAE) and PVI plus left atrial ablation (LAA). There has been considerable debate about the treatment of chronic AF with drugs or radiofrequency catheter ablation.

In order to compare the therapeutic effects of drug therapy and radiofrequency catheter ablation, as well as compare different procedures of radiofrequency catheter ablation, papers published in China and elsewhere between January 1, 2002 and May 1, 2012 concerning the treatment of chronic AF with drug or radiofrequency catheter ablation were retrieved and then analyzed with the RevMan 5.1 software package. This study discusses different strategies for the treatment of chronic AF.

## Materials and methods

### Paper retrieval

Papers published in China and other countries between January 1, 2002 and May 1, 2012, which reported the success rates and relapse rates of drug therapy and catheter ablation for the treatment of chronic AF were retrieved. Databases used were Chinese HowNet, VIP, Wanfang, Medline, Wiley, SpringerLink, Google Scholar and Science Direct.

The search items included the title, keyword and abstract. The following English search terms and the corresponding Chinese terms were used: atrial fibrillation, ablation/catheter ablation, drugs/anti-arrhythmia and chronic atrial fibrillation/permanent atrial fibrillation.

### Inclusion and exclusion criteria

Inclusion criteria were as follows: i) papers published in China and other countries between January 1, 2002 and May 1, 2012; ii) randomized controlled trials (RCTs); iii) patients with chronic AF; iv) clear diagnostic criteria: drugs did not effectively maintain sinus rhythm or persistent AF for >7 days repeated within 6 months; v) data collection with a scientific method; vi) data analysis with a correct and scientific method; vii) interventions including radiofrequency catheter ablation and drug therapy; and viii) only one paper selected from several papers about the same population. Exclusion criteria were as follows: i) non-RCT; ii) data collection with a non-scientific method; iii) data analysis with non-scientific method; iv) literature reviews and v) repeated papers.

### Quality evaluation

The quality of papers was evaluated according to the quality evaluation criteria described in v.4.2.2 of the Cochrane System Assessment handbook. The quality of papers was divided into grades A, B and C based on the randomized method, hidden method, double-blind method, loss of follow-up and exodus of patients from the study. Grade A had low bias and the lowest possibility of bias, and completely conformed to the four quality standards. The four quality standards include randomized method, hidden method, double-blind method, and loss of follow-up and exodus of patients. Grade B had moderate bias and a moderate possibility of bias, and partially conformed to ≥1 quality standards. Grade C had high bias and a high possibility of bias, and did not conform to ≥1 quality standards completely.

### Statistical analysis

According to the requirements of the meta-analysis, data processing was performed and a database was established. Data analysis was performed with the RevMan 5.1 software package. The therapeutic effects of catheter ablation and drug therapy for chronic AF were analyzed, with the odds ratio (OR) as an effective index, and the OR and 95% confidence interval (CI) were calculated. Specific steps were as follows: i) OR served as the summary statistic; ii) an homogeneity test was performed using the χ^2^ test. If P>0.1, multiple independent studies had homogeneity and OR was calculated with the fixed effect model. If P≤0.1, multiple independent studies had heterogeneity and after sensitivity or stratified analyses, the data had homogeneity; then OR was calculated with the fixed effect model. Otherwise OR was calculated with the random effect model; iii) the probability value of the summary statistic was first obtained with the U test. If P≤0.05 was considered to indicate a statistically significant difference; iv) the publication bias was identified with funnel plots. The funnel plots were generated by the RevMan 5.1 software package, with OR values as the x-axis and with SE (log OR) as the y-axis. The publication bias was evaluated by observing whether the funnel plot was symmetrical.

## Results

### Paper retrieval

A total of 20 papers (10–29) were used in this study. Of the 20 papers, eight compared drug therapy with radiofrequency catheter ablation; five compared PVAI and PVAI + CFAE, of which two papers also compared PVAI alone and CFAE alone; four compared PVI + LAA and PVI; and three compared PVI and CPVI (Table I).

### Comparison of success rates between catheter ablation and drug therapy

There were eight papers (10–17) that compared the success rates of catheter ablation and drug therapy. These papers included a total of 951 patients, with catheter ablation as the test group and drug therapy as the control group (Table II).

**Figure 1.**
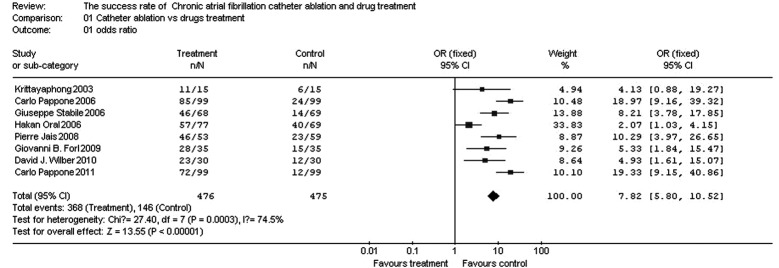
Comparison of success rates between catheter ablation and drug therapy.

In the eight papers, there were 476 patients in the test group and 475 patients in the control group. The homogeneity test (χ^2^=30.58, v=7, P<0.0001) demonstrated that the eight papers had heterogeneity; therefore, the random effect model was adopted. The OR value was 8.94 (95% CI, 4.70–17.02; z=6.68; P<0.0001), suggesting that the success rate was significantly higher for catheter ablation compared with that for drug therapy (Fig. 1).

### Comparison of relapse rates between catheter ablation and drug therapy for treatment of chronic AF

There were seven papers (10,11,13–17) that compared the relapse rates of catheter ablation and drug therapy for treatment of chronic AF. The seven papers included a total of 753 patients, with catheter ablation as the test group and drug therapy as the control group (Table III).

**Figure 2.**
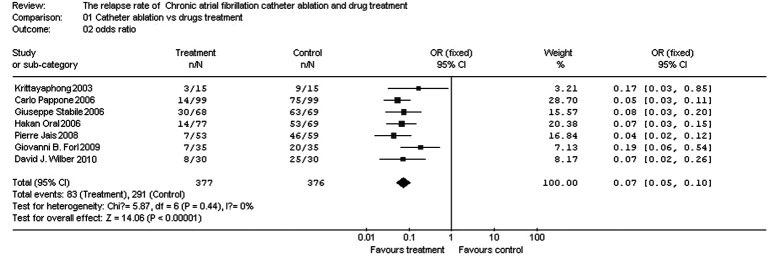
Comparison of relapse rates between catheter ablation and drug therapy.

In the seven papers, there were 377 patients in the test group and 376 patients in the control group. The homogeneity test (χ^2^=5.87, v=6, P=0.44, P>0.10) demonstrated that the seven papers had homogeneity; therefore, the fixed effect model was adopted. The OR value was 0.07 (95% CI, 0.05–0.10; z=14.06; P<0.0001), suggesting that the relapse rate was significantly lower for catheter ablation compared with that for drug therapy (Fig. 2).

### Comparison of success rates between PVAI and CFAE

There were two papers (18,19) comparing the success rates of PVAI and CFAE for treatment of chronic AF. The two papers included a total of 128 patients, with PVAI as the test group and CFAE as the control group (Table IV).

**Figure 3.**
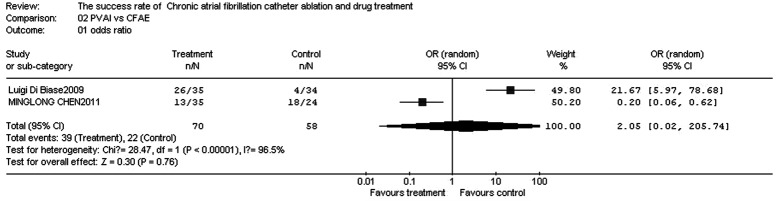
Comparison of success rates between PVAI and CFAE. PVAI, pulmonary vein antrum isolation; CFAE, complex fractional atrial electrogram.

In the two papers, there were 70 patients in the test group and 58 patients in the control group. The homogeneity test (χ^2^=28.47, v=1, P=0.0000, P<0.10) demonstrated that the two papers did not have homogeneity; therefore, the random effect model was adopted. The OR value was 2.05 (95% CI, 0.06–205.74; z=0.30; P=0.76), suggesting that there was no significant difference in success rates between PVAI and CFAE for treatment of chronic AF (Fig. 3).

### Comparison of success rates between PVA I and PVAI + CFAE

There were five papers (18–22) comparing the success rates of PVAI and PVAI + CFAE for treatment of chronic AF. The five papers included a total of 559 patients, with PVAI as the test group and PVAI + CFAE as the control group (Table V).

**Figure 4.**
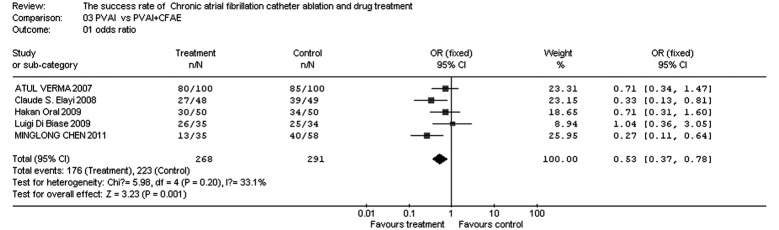
Comparison of success rates between PVAI and PVAI + CFAE for the treatment of chronic AF. PVAI, pulmonary vein antrum isolation; CFAE, complex fractionated atrial electrogram; AF, atrial fibrillation.

In the five papers, there were 268 patients in the test group and 291 patients in the control group. The homogeneity test (χ^2^=5.98, v=4, P=0.20, P>0.10) demonstrated that the papers had homogeneity; therefore, the fixed effect model was adopted. The OR value was 0.53 (95% CI, 0.37–0.78; z=3.23; P=0.001), suggesting that the success rate for treatment of chronic AF was significantly higher for PVAI + CFAE than for PVAI (Fig. 4).

### Comparison of success rates between PVI and PVI + LAA

There were four papers (23–26) in which the success rates of PVI and PVI + LAA in the treatment of chronic AF were compared. These papers included a total of 322 patients, with PVI + LAA as the test group and PVI as the control group (Table VI).

**Figure 5.**
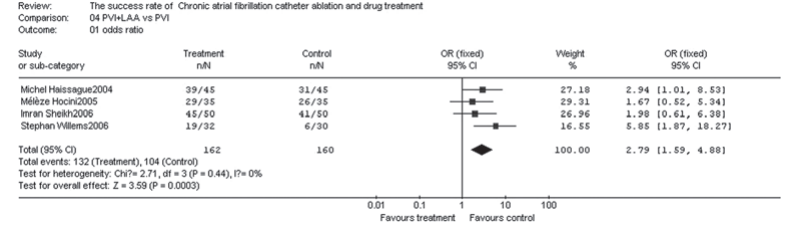
Comparison of success rates between PVI + LAA and PVI. PVI, pulmonary vein isolation; LAA, left atrial ablation.

In the four papers, there were 162 patients in the test group and 160 patients in the control group. The homogeneity test (χ^2^=2.71, v=3, P=0.44, P>0.10) demonstrated that the papers had homogeneity; therefore, the fixed effect model was adopted. The OR value was 2.79 (95% CI, 1.59–4.88; z=3.59; P=0.0003), suggesting that the success rate was significantly higher for PVI + LAA compared with that for PVI (Fig. 5).

### Comparison of success rates between PVI and CPVI

There were three papers (27–29) in which the success rates of PVI and CPVI were compared. The three papers included a total of 310 patients, with PVI as the test group and CPVI as the control group (Table VII).

**Figure 6.**
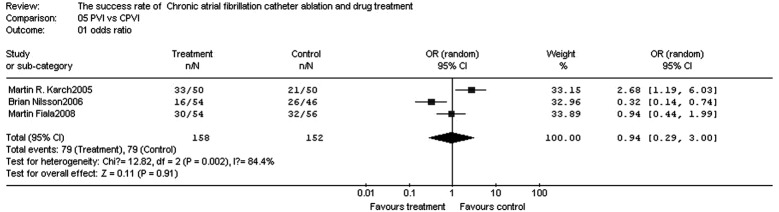
Comparison of success rates between PVI and CPVI. PVI, pulmonary vein isolation; CPVI, circumferential pulmonary vein isolation.

In the three papers, there were 150 patients in the test group and 160 patients in the control group. The homogeneity test (χ^2^=12.82, v=2, P=0.002, P<0.10) demonstrated that the papers did not have homogeneity; therefore, the random effect model was adopted. The OR value was 0.94 (95% CI, 0.29–3.00; z=0.11; P=0.91), suggesting that there were no significant differences in the success rates between PVI and CPVI for treatment of chronic AF (Fig. 6).

### Publication bias

All funnel plots comparing the success rates of catheter ablation and drug therapy, PVAI and CFAE, PVAI and PVAI + CFAE, PVI + LAA and PVI, and PVI and CPVI were essentially symmetrical, and the majority of the points were located within the 95% CI. The funnel plot comparing the relapse rates of catheter ablation and drug therapy was essentially symmetrical and the majority of the points were located within the 95% CI.

## Discussion

In this study, a meta-analysis of catheter ablation and drug therapy for the treatment of chronic AF was performed according to Preferred Reporting Items for Systematic Reviews and Meta-Analyses (PRISMA). The results indicated that for the treatment of chronic AF, the success rate is higher and the relapse rate is lower for catheter ablation compared with that for drug therapy. There were no significant differences between the success rates of PVAI and CFAE, and PVI and CPVI; however, the success rates were higher for PVAI + CFAE compared with that for PVAI, and for PVI + LAA compared with that for PVI. The results of publication bias indicated that the results of the meta-analysis were stable and reliable, truly reflecting the status of catheter ablation and drug treatment of chronic AF.

AF treatment includes rate control and rhythm control. Although it has been reported that rate control is a reasonable choice for the treatment of AF (30), rate control alone does not reduce the risk of cerebral apoplexy and improve atrioventricular synchrony. Antithrombotic therapy with warfarin decreases the incidence of cerebral apoplexy and reduces the mortality rate; however, there is the risk of bleeding, which requires long-term monitoring of international normalized ratio (INR) of prothrombin time with low patient compliance. Compared with rate control, rhythm control decreases the mortality rate and the incidence of transient ischemic attack (TIA), cerebral infarction (31), systemic embolism, hemorrhea and heart failure (32). Therefore, rhythm control is likely to be more effective than rate control. The conventional methods of rhythm control include anti-arrhythmic drugs, direct current countershock and the surgical maze procedure. However, these methods are limited in clinical practice due to their therapeutic effects and safety. In the past 20 years, a great deal of attention has been paid to catheter ablation for the treatment of AF. In the AF treatment guidelines established by ACC/AHA/ESC in 2008, catheter ablation is suitable for patients who exhibit no therapeutic effects following treatment with class I or III anti-arrhythmic drugs, are unable to tolerate the side-effects of drugs or have symptomatic heart failure or low cardiac output prior to the use of anti-arrhythmic drugs.

The pathogenesis of AF is not completely clear. At present, its mechanism mainly includes triggering factors and an electrical substrate (or atrial substrate). The majority of the triggering factors are located in the pulmonary veins and superior vena cava; however, a few triggering factors are located in the crista terminalis, coronary sinus, Marshall ligaments and atrial posterior wall. Triggering factors are also called triggering foci. Electrical substrate refers to the changes in electrophysiological characteristics to maintain AF and mainly includes electrical reconstitution, anatomical reconstitution, reconstitution of autonomic nerves and reconstitution of the renin-angiotensin system. Atrial dilatation, myocarditis, myocardial fibrosis and increased autonomic nervous tension all serve as triggering factors and/or an electrical substrate to lead to AF. Triggering factors and the electrical substrate may be located in the same place or at different locations. Radiofrequency ablation is used to treat AF through the isolation of triggering factors and interference with the electrical substrate (33,34).

The limitations of the current study are that only a small number of papers were included in the study, unpublished papers from conferences were not included and the quality control standards were not completely uniform. Future meta-analyses should include a greater number of RCTs.

Our results suggest that catheter ablation is more effective than drug therapy in the treatment of AF. There are no significant differences in success rates between two single procedures for catheter ablation; however, the success rate is higher in PVAI + CFAE compared with that in PVAI, and in PVI + LAA compared with that in PVI.

## Figures and Tables

**Table I.** Original papers included in this meta-analysis.

No. (ref.)	Publication date (year)	First author	Title of paper
1 (10)	2010	David J. Wilber	Comparison of antiarrhythmic drug therapy and radiofrequency catheter ablation in patients with paroxysmal atrial fibrillation: a randomized controlled trial
2 (11)	2006	Carlo Pappone	A randomized trial of circumferential pulmonary vein ablation versus antiarrhythmic drug therapy in paroxysmal atrial fibrillation: the APAF study
3 (12)	2011	Carlo Pappone	Radiofrequency catheter ablation and antiarrhythmic drug therapy: a prospective, randomized, 4-year follow-up trial: the APAF study
4 (13)	2003	R. Krittayaphong	A randomized clinical trial of the efficacy of radiofrequency catheter ablation and amiodarone in the treatment of symptomatic atrial fibrillation
5 (14)	2009	Giovanni B. Forleo	Catheter ablation of atrial fibrillation in patients with diabetes mellitus type 2: results from a randomized study comparing pulmonary vein isolation versus antiarrhythmic drug therapy
6 (15)	2008	Pierre Jaïs	Catheter ablation versus anti-arrhythmic drugs for atrial fibrillation: the A4 study
7 (16)	2006	Hakan Oral	Circumferential pulmonary-vein ablation for chronic atrial fibrillation
8 (17)	2006	Giuseppe Stabile	Catheter ablation treatment in patients with drug-refractory atrial fibrillation: a prospective, multi-centre, randomized, controlled study (Catheter Ablation For The Cure Of Atrial Fibrillation Study)
9 (18)	2009	Luigi Di Biase	Atrial fibrillation ablation strategies for paroxysmal patients: randomized comparison between different techniques
10 (19)	2011	Minglong Chen	Randomized comparison between pulmonary vein antral isolation versus complex fractionated electrogram ablation for paroxysmal atrial fibrillation
11 (20)	2008	Claude S. Elayi	Ablation for longstanding permanent atrial fibrillation: results from a randomized study comparing three different strategies
12 (21)	2009	Hakan Oral	A randomized assessment of the incremental role of ablation of complex fractionated atrial electrograms after antral pulmonary vein isolation for long-lasting persistent atrial fibrillation
13 (22)	2007	Atul Verma	Efficacy of adjuvant anterior left atrial ablation during intracardiac echocardiography-guided pulmonary vein antrum isolation for atrial fibrillation
14 (23)	2006	Stephan Willems	Substrate modification combined with pulmonary vein isolation improves outcome of catheter ablation in patients with persistent atrial fibrillation: a prospective randomized comparison
15 (24)	2005	Mélèze Hocini	Techniques, evaluation, and consequences of linear block at the left atrial roof in paroxysmal atrial fibrillation: a prospective randomized study
16 (25)	2004	Michel Haïssaguerre	Changes in atrial fibrillation cycle length and inducibility during catheter ablation and their relation to outcome
17 (26)	2006	Imran Sheikh	Pulmonary vein isolation and linear lesions in atrial fibrillation ablation
18 (27)	2008	Martin Fiala	Pulmonary vein isolation using segmental versus electro-anatomical circumferential ablation for paroxysmal atrial fibrillation: over 3-year results of a prospective randomized study
19 (28)	2005	Martin R. Karch	Freedom from atrial tachyarrhythmias after catheter ablation of atrial fibrillation: a randomized comparison between 2 current ablation strategies
20 (29)	2006	Brian Nilsson	Recurrence of pulmonary vein conduction and atrial fibrillation after pulmonary vein isolation for atrial fibrillation: a randomized trial of the ostial versus the extraostial ablation strategy

**Table II.** Papers comparing the success rates of catheter ablation and drug therapy for treatment of chronic atrial fibrillation.

Number	Reference	Publication date (year)	First author	Test group (n)		Control group (n)	
	
				Success	Total	Success	Total
1	10	2010	David J. Wilber	23	30	12	30
2	11	2006	Carlo Pappone	72	99	12	99
3	12	2011	Carlo Pappone	85	99	24	99
4	13	2003	R. Krittayaphong	11	15	6	15
5	14	2009	Giovanni B. Forleo	28	35	15	35
6	15	2008	Pierre Jaïs	46	53	23	59
7	16	2006	Hakan Oral	57	77	40	69
8	17	2006	Giuseppe Stabile	46	68	14	69

**Table III.** Papers comparing the relapse rates of catheter therapy and drug therapy.

Number	Reference	Publication date (year)	First author	Test group (n)		Control group (n)	
	
				Relapse	Total	Relapse	Total
1	10	2010	David J. Wilber	8	30	25	30
2	11	2006	Carlo Pappone	14	99	75	99
3	13	2003	R. Krittayaphong	3	15	9	15
4	14	2009	Giovanni B. Forleo	7	35	20	35
5	15	2008	Pierre Jaïs	7	53	46	59
6	16	2006	Hakan Oral	14	77	53	69
7	17	2006	Giuseppe Stabile	30	68	63	69

**Table IV.** Papers comparing the success rates of PVAI and CFAE.

Number	Reference	Publication date (year)	First author	Test group (n)		Control group (n)	
	
				Success	Total	Success	Total
1	18	2009	Luigi Di Biase	26	35	4	34
2	19	2011	Minglong Chen	13	35	18	24

PVAI, pulmonary vein antrum isolation; CFAE, complex fractionated atrial electrogram.

**Table V.** Papers comparing the success rates of PVAI and PVAI + CFAE.

Number	Reference	Publication date (year)	First author	Test group (n)		Control group (n)	
	
				Success	Total	Success	Total
1	18	2009	Luigi Di Biase	26	35	25	34
2	19	2011	Minglong Chen	13	35	40	58
3	20	2008	Claude S. Elayi	27	48	39	49
4	21	2009	Hakan Oral	30	50	34	50
5	22	2007	Atul Verma	80	100	85	100

PVAI, pulmonary vein antrum isolation; CFAE, complex fractionated atrial electrogram.

**Table VI.** Papers comparing the success rates of PVI + LAA and PVI.

Number	Reference	Publication date (year)	First author	Test group (n)		Control group (n)	
	
				Success	Total	Success	Total
1	14	2006	Stephan Willems	19	32	6	30
2	15	2005	Mélèze Hocini	39	45	31	45
3	16	2004	Michel Haïssaguerre	29	35	26	35
4	17	2006	Imran Sheikh	45	50	41	50

PVI, pulmonary vein isolation; LAA, left atrial ablation.

**Table VII.** Papers comparing the success rates of PVI and CPVI.

Number	Reference	Publication date (year)	First author	Test group (n)		Control group (n)	
	
				Success	Total	Success	Total
1	27	2008	Martin Fiala	30	54	32	56
2	28	2005	Martin R. Karch	33	50	21	50
3	29	2006	Brian Nilsson	26	46	16	54

PVI, pulmonary vein isolation; CPVI, circumferential pulmonary vein isolation.
